# Forming a stone in pelviureteric junction obstruction: cause or effect?

**DOI:** 10.1590/S1677-5538.IBJU.2015.0515

**Published:** 2017

**Authors:** Theodora Stasinou, Andreas Bourdoumis, Junaid Masood

**Affiliations:** 1 South Manchester University Hospitals NHS Foundation Trust, Manchester, UK;; 2 North Manchester General Hospital, Acute Pennine Hospitals NHS Trust, Manchester, UK;; 3 Homerton University Hospital NHS Foundation Trust, London, UK

**Keywords:** Pelviureteric Junction Obstruction, Calculi, Urolithiasis, Nephrostomy, Percutaneous

## Abstract

**Objectives:**

To investigate a possible causal relationship for stone formation in pelviureteric junction obstruction and to outline management options.

**Materials and Methods:**

A literature search and evidence synthesis was conducted via electronic databases in the English language using the key words pelviureteric junction obstruction; urolithiasis; hyperoxaluria; laparoscopic pyeloplasty; flexible nephroscopy; percutaneous nephrolithotomy, alone or in combination. Relevant articles were analysed to extract conclusions.

**Results:**

Concomitant pelviureteric junction obstruction (PUJO) and renal lithiasis has been reported only scarcely in the literature. Although PUJO has been extensively studied throughout the years, the presence of calculi in such a patient has not received equal attention and there is still doubt surrounding the pathophysiology and global management.

**Conclusions:**

Metabolic risk factors appear to play an important role, enough to justify metabolic evaluation in these patients. Urinary stasis and infection are well known factors predisposing to lithiasis and contribute to some extent. The choice for treatment is not always straightforward. Management should be tailored according to degree of obstruction, renal function, patient symptoms and stone size. Simultaneous treatment is feasible with the aid of minimally invasive operative techniques and laparoscopy in particular.

## INTRODUCTION

Pelviureteric junction obstruction (PUJO) is well described in the literature as far as diagnosis and treatment are concerned. Yet, there is much controversy regarding stone formation and management in these patients. PUJO was first described as a syndrome by Dietl in 1864 (1) and the ensuing fibrotic changes were demonstrated by Allen TD in 1970 (2). Subsequently, it was proven that if left untreated the narrow junction eventually leads to deterioration of renal function in the majority of cases (3, 4). PUJO is classified as primary (congenital or intrinsic) when dysfunctional smooth muscle and excess collagen deposition leads to hydronephrosis with clockwise rotation of the renal pelvis and a high ureteral origin (4-7). It also occurs commonly as a secondary (acquired or extrinsic) abnormality, where a crossing vessel (i.e. lower pole artery), a fibrous band or other disease (retroperitoneal fibrosis, renal cysts, xanthogranulomatous pyelonephritis, malignancy) lead to obstruction by compression and kinking at the junction (8, 9). Concomitant lithiasis of the urinary tract is not uncommon and whether it co-exists as a separate entity or is the result of a narrow renal outflow tract is still debated. The prevalence of lithiasis in patients with malformations of the kidney is described as higher than that of the general population (10). In a retrospective review of 1639 paediatric patients during a 45 year period at the Mayo Clinic, the prevalence was 70-fold that of the aged matched population (11, 12). This seems to be also true for the adult population (13). In an early series, David and Lavengood (14) reported concomitant lithiasis in 16% of patients undergoing open pyeloplasty, whereas others reported an incidence of up to 20% (15). PUJO in horseshoe kidneys is described as high as 35% (16) and Lampel et al. suggested that at least 14% of stones treated in such patients were associated with a narrow pelviureteric junction (17).

We have conducted a literature search in three-3-electronic databases (Medscape/E-medicine, Pub Med, EmBase) using the following key words: pelviureteric junction obstruction; urolithiasis; hyperoxaluria; laparoscopic pyeloplasty; flexible nephroscopy; percutaneous nephrolithotomy, alone or in combination. We isolated articles in the English language, relevant to research and/or reports of concomitant lithiasis on a background of PUJO. Overall, 17 articles were identified, mostly case series and presentation of surgical techniques. Only two reports (11, 13) focused on identifying any underlying pathophysiological changes in paediatric populations, while one further study examined the metabolic factors in renal stones coinciding with PUJO (18).

### Pathogenesis of calculi in PUJO

There are few reports in the literature that examine the significance and/or correlation of the ultrastructural changes in the narrow pelviureteric junction with the incidence of renal calcul (11-13). In one such retrospective analysis, all patients had histologic evidence of tissue changes (increased fibrosis) associated with anatomical obstruction, similar to those originally described by Allen TD for true congenital PUJO (13). In theory, an impacted stone at the pelviueteric junction is likely to produce local inflammation and edema sufficient to create circumstances similar to PUJO or it may provoke an inflammatory reaction severe enough to produce a stricture, but strong evidence are sparse (9, 19). It is quite difficult to differentiate between the two at the time of endourological stone treatment and a wise approach is to defer further intervention until more imaging and investigations become available. Another hypothesis is that a delayed washout due to the junction results in crystal agglomeration and nucleation that eventually develop into calculi (20). Whether stones in patients with PUJO have an underlying metabolic causative factor (14, 15) or represent the result of the anatomical condition per se (20) remains an area of controversy. It has been shown that urinary stasis does not appear to be the sole contributor to lithiasis in horseshoe kidneys, and that urinary tract infection and metabolic factors play an important and synergistic role (10). Further evidence from retrospective and prospective studies suggest that urinary stasis may, in fact, have little to do with the pathogenesis of renal stones in PUJO. In their retrospective study, Husmann et al. reviewed medical records of 111 patients who underwent pyeloplasty and simultaneous stone removal with a median follow-up of 10 years. Interestingly, a significant percentage of the study group (n=34, 31%) presented with infectious struvite stones (magnesium-ammonium-sulphate), prompting the authors to sub classify outcomes into struvite and non-struvite groups. In total, 36 patients (32.5%) presented with increased concentrations of several known lithogenic substances in preoperative meatbolic evaluation. The incidence of this finding in these patients was similar to that found in idiopathic stone formers. The abnormality consisted of varying levels of hypercalciuria, hyperoxaluria, hyperuricosuria and hypocitraturia (Table-1). In this cohort, all patients with primary hyperparathyroidism had hypercalciuria and all patients with distal renal tubular acidois had hypercalciuria and hypocitraturia. Long term follow-up of the nonstruvite group (n=53) treated by observation alone yielded a 55% stone recurrence rate, with a median interval to recurrence of 9.5 years. Subsequent metabolic evaluation in this group revealed that 83% had an underlying abnormality. In contrast, subsequent medical management in the treatment arm (n=24) yielded only 17% stone recurrence rate. In the struvite group, 43% of recurrent calculi occurred in the contralateral kidney. Long term antibiotic treatment appeared to be beneficial with regards to stone recurrence in this group. The same authors subsequently reviewed a paediatric population with similar characteristics in retrospect, and found a recurrence rate of 68% in long term follow-up, with comparable results as for the metabolic factors found in adults, further supporting the concept of an underlying metabolic etiology (11).

**Table 1:** Metabolic risk factors for stone formation in patients with PUJO (percentages correspond to those that were metabolically evaluated).

Risk factors Study Series	Hyperoxaluria	Hypercalciuria	Hyperuricosuria	Hypocitraturia
Hussman et al. (13)	N/A	61%(observation group) and 17%(struvite group)	11% (observation group) and 8%(struvite group)	22%(observation group) and 8% (struvite group)
Hussman et al. (11)	N/A	36% (observation group) and 17% (struvite group)	14% (observation group) and 17%(struvite group)	9% ( non-struvite only)
Matin and Streem (18)	24% vs 12% in control	33% vs 12% in control	29% vs 8% in control	19% vs 27% in control

In their prospective observational study, Matin and Streem evaluated 47 patients with congenital PUJO for factors predisposing to lithiasis (18). Of the 21 patients with stones, 67% presented identifiable metabolic risk factors vs. 38% of the 26 control patients with PUJO and no stones. The incidence was not unlike that found in stone forming populations (18, 20). The composition of such stones was found to be calcium oxalate in 93% of patients, with or without calcium phosphate as an additional mineral. The authors acknowledge small number of patients in the study (n=47), but pertain to the prospective design of the evaluation, to conclude that metabolic evaluation is required in the treatment plan of concurrent PUJO and renal calculi. The same conclusion is also produced by Hussman (Table-1) (13). Hyperoxaluria and hypercalciuria have been confirmed as having positive correlation with PUJO and lithiasis in respective series of paediatric patients (21, 22). Summary of the metabolic risk factors identified during these studies is presented in Table-1. In the retrospective study by Bernado et al., 90 patients with PUJO who underwent endopyelotomy and simultaneous stone removal were compared with 80 patients without obstruction who underwent only stone extraction. The authors argue against metabolic factors as a prerequisite, since 71.4% of patients without PUJO were found to have a metabolic abnormality that predisposed to urinary stones, as opposed to 19% with obstruction (23). The authors concluded that correction of the anatomic obstruction facilitates the drainage of urine, thus decreasing the incidence of recurrent urinary stone formation.

Overall, the available evidence point toward a combination of factors that seem to be responsible for lithiasis in PUJO other than urinary stasis caused by the obstruction. An undiagnosed metabolic abnormality and probably genetic predisposition are likely, while urinary tract infection and pH appear to play a part as well. It is therefore important to consider these possibilities and include respective appropriate measures in the formulation of a global treatment strategy.

### Management options

**Figure 1:**
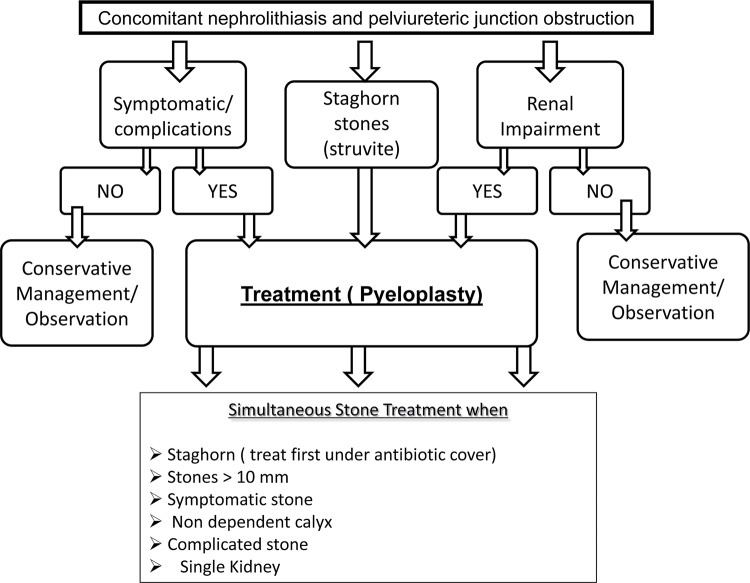
Suggested algorithm for the management of established PUJO and concomitant nephrolithiasis..

In order to adequately manage patients with PUJO and renal stones, one is guided by answering two important questions, that of when and how to treat. The significance of the exact location and number of the calculi in the pelvicalyceal system is not adequately described in the majority of the studies. An initial period of observation seems reasonable for asymptomatic stones of less than 5mm in greatest dimension, accompanied by regular follow-up of the degree of obstruction and renal function (24). With increasing symptoms, stone size, deteriorating renal function and/or recurrent infections, active treatment becomes necessary. Minimally invasive procedures should be preferred where available. While open, laparoscopic and lately robotically assisted pyeloplasty constitute established treatment options for PUJO, no such consensus exists for treating the stone. For our proposed algorithm in Figure-1, we suggest that PUJO is an already established diagnosis at the time of choice of treatment, preferable by nuclear renogram studies that demonstrate obstruction as part of the pre-operative assessment. In the modern era of endoscopic stone surgery simultaneous treatment appears feasible, even in cases with multiple stones and difficult anatomy, i.e. calyceal stones, where retrograde flexible instruments seem to be very useful (25-28). Table-2 provides a summary of the existing evidence in endoscopic management of such cases. A recently published review by Skolarikos et al. and also laparoscopic series support this concept (29-34). Endoscopic combined intrarenal surgery (ECIRS) could also be an option, although the evidence is lacking. For staghorn and struvite stones in particular, it is prudent to take caution and ensure peri-and postoperative antibiotic cover guided by urine culture and local sensitivity patterns (35, 36). The duration and programming of follow-up is yet to be determined, but should include a history of symptoms, routine renal biochemistry and radionuclide imaging, i.e. MAG-3 renogram as a minimum (37, 38). We also recommend performing a thorough metabolic work-up, similar to that proposed for recurrent stone formers, both before and after definitive treatment, in order to identify the metabolic stone formers and formulate an appropriate preventive strategy (39).

**Table 2:** Summary of results following simultaneous PUJO correction and stone treatment.

Study (retrospective series)	PUJO Treatment	Stone Treatment	PUJO reversal	Stone Free Rate (%)	Mean follow-up (months)
Cassis et al. (33)	Antegrade endopyelotomy	PCNL	89%	100%	12
Ramakumar et al. (34)	Laparoscopic pyeloplasty	Flexible cystoscopy/ Pyelolithotomy	90%	80%	12
Ball et al. (27)	Laparoscopic pyeloplasty	Flexible Nephroscope	100%	85%	8.5
Wheelan et al. (28)	Laparoscopic pyeloplasty	Flexible Ureterosocope	90%	100%	13
Agarwal et al. (40)	Laparoscopic pyeloplasty	PCNL	50% (partial)	100%	12
Shrivastava et al. (25)	Laparoscopic pyeloplasty	Flexible cystoscope/ ureteroscope	90%	75%	34
Berkman et al. (26)	Antegrade /Retograde endopyelotomy	Flexible nephroscope/ ureteroscope	71% /90%	N/A	25-29

## CONCLUSIONS

Stone disease in pelviureteric junction obstruction is associated with an underlying metabolic disorder in up to a third of patients. Metabolic risk factors appear to play an important role, enough to justify metabolic evaluation of such patients. Urinary stasis and infection are well known factors predisposing to lithiasis and also appear to be contributory factors. The choice for treatment is not always straightforward and relies on several factors, including organization of the department with dedicated stone clinic services, availability of appropriate equipment to carry out complex endourological surgery and experience in postoperative follow-up and complication management. Upon verification of PUJO with nuclear functional imaging studies, further intervention should be tailored according to degree of obstruction, renal function, patient symptoms and stone burden. Simultaneous treatment is feasible with the aid of minimally invasive operative techniques and laparoscopic approach in particular appears to be the most promising solution (Table-2). Robotically-assisted laparoscopy is rapidly growing in the field and appears promising (40). Metabolic evaluation should be an integral part of initial evaluation as well as follow-up and form the basis for future preventative planning against recurrences.
